# Anti-glomerular basement membrane disease superimposed on membranous nephropathy: a case report and review of the literature

**DOI:** 10.1186/1752-1947-4-237

**Published:** 2010-08-02

**Authors:** Dhruval Patel, Noel Nivera, Allan R Tunkel

**Affiliations:** 1 Department of Internal Medicine, Monmouth Medical Center, Long Branch, NJ, USA; 2 Department of Medicine, Monmouth Medical Center, 300 Second Avenue, Long Branch, NJ 07740, USA

## Abstract

**Introduction:**

Anti-glomerular basement membrane disease is a rare autoimmune disorder characterized by pulmonary hemorrhage, crescentic glomerulonephritis and the presence of circulating anti-glomerular basement membrane antibodies. The simultaneous occurrence of both anti-glomerular basement membrane disease and membranous nephropathy is rare.

**Case presentation:**

A 59-year-old Hispanic man presented with acute onset of nausea and vomiting and was found to have renal insufficiency. Work-up included a kidney biopsy, which revealed anti-glomerular basement membrane disease with underlying membranous nephropathy. He was treated with emergent hemodialysis, intravenous corticosteroids, plasmapheresis, and cyclophosphamide without improvement in his renal function.

**Conclusion:**

Simultaneous anti-glomerular basement membrane disease and membranous nephropathy is very rare. There have been 16 previous case reports in the English language literature that have been associated with a high mortality and morbidity, and a very high rate of renal failure resulting in hemodialysis. Co-existence of membranous nephropathy and anti-glomerular basement membrane disease may be immune-mediated, although the exact mechanism is not clear.

## Introduction

Anti-glomerular basement membrane (anti-GBM) disease is a rare autoimmune disorder with significant morbidity and mortality and is characterized by pulmonary hemorrhage, crescentic glomerulonephritis, and the presence of circulating anti-GBM antibodies which bind to the alpha-3 chain of type 4 collagen found in the glomerular and alveolar basement membranes [1]. The etiology of anti-GBM disease is unclear, but may result from hydrocarbon exposure; specific HLA molecules have also been found to be associated with disease. Anti-GBM disease accounts for approximately 10 to 20 percent of patients with rapidly progressive crescentic glomerulonephritis in the United States. The diagnosis is established by demonstration of high titers of anti-GBM antibodies in the circulation and/or renal biopsy. Early treatment with high-dose corticosteroids, plasmapheresis and cyclophosphamide is recommended because early and appropriate treatment may reverse the extent of renal damage and potentially prevent the need for life-long dialysis. In untreated patients, anti-GBM disease progresses to renal failure and death. There have been very few case reports documenting the simultaneous appearance of anti-GBM disease and membranous nephropathy. Here, we present a case of combined disease, and review the literature on this important, but rare, condition.

## Case presentation

A 59-year-old Hispanic man with no known significant past medical history was well until two weeks prior to presentation when he noted some chest congestion and early morning nausea. One week later, he presented to a local walk-in clinic where he was treated with hydrocodone, chlorpheniramine (Tussionex), and ciprofloxacin without improvement. Five days later, he presented to our Emergency Department with complaints of nausea, vomiting, fatigue, decreased urination, and generalized malaise. He denied hemoptysis, hematuria, rash, or use of non-steroidal anti-inflammatory agents. He believed that he had received a blood transfusion during the Vietnam War secondary to bleeding after a forearm injury. Physical examination revealed that his temperature was 98.2°F, pulse 88 per minute, respiratory rate 20 per minute, and blood pressure 160/78 mmHg. Examination was otherwise unremarkable.

**Figure 1:**
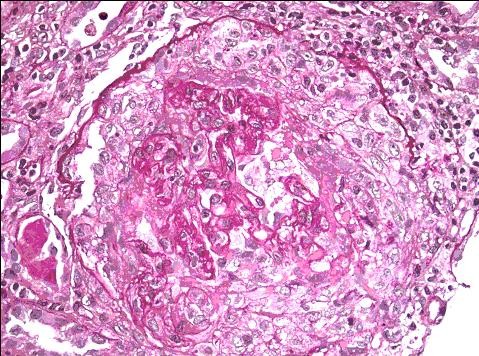
**Light microscopy revealed cellular crescents in all sampled 20 glomeruli with extensive fibrinoid necrosis and neutrophil margination**.

**Figure 2:**
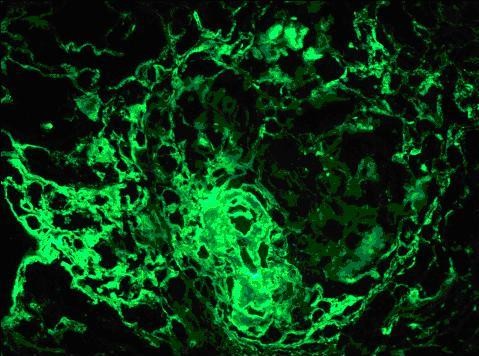
**Immunofluorescence findings of linear glomerular capillary wall positivity of 3 + intensity for IgG with 1-2+ C3, 3+ kappa and 2+ lambda support the diagnosis of anti GBM disease**.

**Figure 3:**
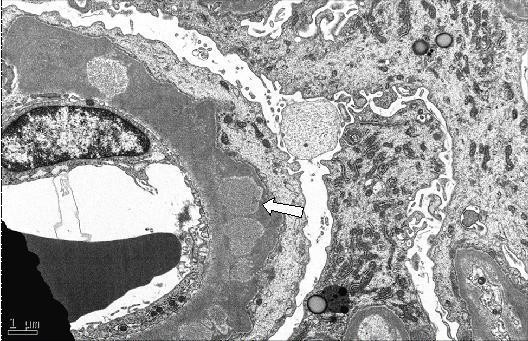
**Electron microscopy showed global subepithelial deposits which is diagnostic for underlying membranous glomerulopathy stage 3**.

Laboratory studies revealed a serum creatinine of 25 mg/dL (normal range 0.6 to 1.2 mg/dL), BUN 175 mg/dL (normal range 5 to 21 mg/dL), hemoglobin 10 gm/dL (normal range 13.5 to 18 gm/dL), and hematocrit 28.9% (normal range 41 to 54%). Urine analysis showed proteinuria of over 300 mg, too numerous to count red blood cells per high power field, and too numerous to count white blood cells per high power field; the urine was negative for eosinophils, red blood cell casts and Bence Jones protein. Serum potassium and phosphorus were 5.0 mg/dL and 9.4 mg/dL, respectively. Serum albumin was 3.1 gm/dL (normal range 3.5 to 5.0 gm/dL). ANA, C-ANCA, P-ANCA, hepatitis panel, and HIV antibody tests were negative. Serum protein electrophoresis, creatine phosphokinase, quantitative Immunoglobin assay, complement levels and chest radiograph were all normal. A renal ultrasound showed that the kidneys were of normal size. He was started on hemodialysis and methylprednisolone one gram intravenously daily. Kidney biopsy revealed cellular crescents in all 20 glomeruli seen on the biopsy specimen (Figure 1). Immunofluorescent staining revealed linear glomerular capillary wall positivity of 3+ intensity for IgG with C3, 3+ kappa and 2+ lambda (Figure 2), supporting the diagnosis of anti-GBM disease. Electron microscopy revealed global subepithelial deposits, diagnostic of membranous glomerulopathy (Figure 3). The serum anti-GBM antibody was positive with titer of over100 units/mL. He was started on plasmapharesis, cyclophosphamide and prednisone, and was maintained on hemodialysis. There was no significant improvement in his renal function. He is being maintained on hemodialysis after six months following his initial presentation. He is currently awaiting kidney transplantation.

## Discussion

This case of co-existing anti-GBM disease and membranous nephropathy is very rare. His biopsy showed 20 out of 20 glomeruli visualized revealing acute and severe cellular crescents on light microscopy. Immunofluoroscence staining revealed linear glomerular capillary wall positivity of 3+ intensity for IgG with 1-2+ C3, 3+ kappa and 2+ lambda, supporting the diagnosis of anti-GBM disease. In addition, electron microscopy revealed subepithelial global deposits, diagnostic of membranous glomerulopathy. The findings of membranous glomerulopathy were chronic and predated the development of anti-GBM disease. He presented with an unusually high serum creatinine of 25 mg/dL, consistent with extensive renal damage at the time of presentation.

A review of the literature revealed 16 previously reported cases. (Table 1) [2-16]. The mean age of the patients was 46 years (range 19 to 65 years), with a predominance of men (11 cases). Four patients had pulmonary involvement in the form of Goodpasture's syndrome at the time of presentation. The mean serum creatinine level at presentation was 8.8 mg/dL (range 0.7 to 25 mg/dL). Eight patients presented with edema, three with hemoptysis, two with cough, two with hematuria, and one patient had fever and a sore throat; our patient presented with nausea and oliguria. The outcome was poor as four patients died and nine patients remained on hemodialysis despite treatment with corticosteroids and plasmapheresis. Only three patients had a good prognosis without the need for hemodialysis; of these patients, the serum creatinine on presentation was 1.2 mg/dL, 3.0 mg/dL and 0.7 mg/dL, indicating that these patients had less renal damage. Five of the patients who initially had membranous nephropathy followed by anti-GBM disease were middle aged to elderly, in contrast to five cases of anti-GBM disease preceding membranous nephropathy in which all were young adults [15]. Savige *et al. *also reported six patients with both anti-GBM disease and membranous nephropathy, all of whom were treated with steroids, cyclophosphomide and plasmapheresis. Five of the six patients were between the ages of 15 to 22 years and recovered well; another patient was 47 years old and remained dialysis dependent [17]. Interestingly, in these younger patients, hematuria and/or hemoptysis were the presenting symptoms, in contrast to the middle age group who initially presented with edema consistent with membranous glomerulonephritis, followed by anti-GBM disease. The proteinuria ranged from trace to 20.7 gm/24 hr, depending on extent of renal damage.

**Table 1:** Characteristics of 17 patients with combined anti-GBM disease and membranous nephropathy.

Case Number [ref #]	Age/Sex	Initial serum creatinine (mg/dL)	Clinical presentation	Treatment	Outcome
1 [2]	49/M	18.0	Edema	Steroids	Died

2 [3]	53/M	13.0	Edema	Steroids, azathioprine	Hemodialysis

3 [3]	44/M	3.1	Edema	Steroids	Hemodialysis

4 [4]	20/F	1.2	Cough, syncope	None	Survived

5 [5]	19/F	10.5	Hemoptysis	Steroids (p)*, azathioprine, nephrectomy	Hemodialysis

6 [6]	25/M	2.1	Hemoptysis	Steroids, PP**	Died

7 [7]	65/M	4.0	Edema	Steroids, PP, cyclophosphamide	Died

8 [8]	20/M	3.0	Fever, sore throat, lumbar pain	Steroids, PP	Survived

9 [9]	54/M	1.1	Cough, arthralgia	Steroids, PP	Hemodialysis

10 [10]	60/M	19.5	Fatigue, anorexia, edema	Steroids (p), PP	Hemodialysis

11 [11]	57/not reported	11.8	Fatigue, hemoptysis	Steroids (p), PP, cyclophosphamide	Hemodialysis

12 [12]	50/M	0.7	Edema	Steroids (p), PP, cyclophosphamide	Survived

13 [13]	50/F	8.9	Edema, nausea	Steroids (p), PP	Hemodialysis

14 [14]	54/F	8.9	Hematuria	Steroids, PP	Hemodialysis

15 [15]	49/M	7.3	Hematuria, flank pain, fatigue	Steroids, PP, cyclophosphamide	Hemodialysis

16 [16]	50/F	11.6	Edema	Steroids, PP, cyclophosphamide	Hemodialysis

17 (present case)	59/M	25.0	Fatigue, oliguria, nausea	Steroids, PP, cyclophosphamide	Hemodialysis

PP, plasmapheresis; p, pulse steroid therapy

Immunologic mechanisms involved in the pathogenesis of anti-GBM disease may directly or indirectly lead to the genesis of membranous-type deposits. It is possible that the intramembranous and epimembranous immune complexes found in membranous nephropathy alter the glomerular basement membrane, causing release of normal or altered glomerular basement material with subsequent development of anti-GBM antibodies and crescentic glomerulonephritis. Induced autoimmune disease in brown Norway rats with mercuric chloride demonstrated initial anti-GBM disease with linear immunoglobulin deposit formation together with formation of autoantibodies directed against multiple basement membrane proteins and proteoglycan components [18]. Membranous deposits may subsequently arise from *in situ *immune complex formation in anti-GBM disease, increasing antigen synthesis by injured podocytes and facilitated by the capping and shedding of antigen-antibody complexes into the subepithelial space. Alternatively, anti-GBM antibodies might arise after glomerular basement membrane damage that occurs as a vasculitis involving the glomerular capillaries; damage to the glomerular basement membrane might uncover "hidden antigens", inducing the formation of antibodies to this membrane. This mechanism may also explain anti-GBM disease after primary glomerulonephritis such as membranous nephropathy or IgA glomerulonephritis [19].

## Conclusion

Anti-GBM disease is a reversible cause of renal failure if diagnosed and treated in the early stages of disease. Recurrence following successful treatment is low and, even in patients who have undergone transplantation, there is a low likelihood of involvement in the transplanted kidney. Anti-GBM disease superimposed on membranous nephropathy is very rare; only 16 cases have been previously reported in the English language literature. Despite treatment with immunosuppressive agents and/or plasmapheresis, most of the patients remained on hemodialysis. Knowing the significant toxicity from treatment with cyclophosphomide and corticosteroids, their role in treatment of patients with anti-GBM disease and concurrent membranous nephropathy in middle-aged patients with higher serum creatinine levels and extensive renal involvement on presentation is debatable and needs more study. Patients who presented with a low serum creatinine, and were younger, were more likely to respond to cytotoxic treatment and have a favorable outcome. Advances in elucidating the structure of the glomerular basement membrane antigen and the identification of the pathogenic B and T cell epitopes, along with new insights into the pathogenetic mechanisms at the cellular and molecular level, will facilitate the development of targeted strategies for prevention, detection, and treatment of human anti-GBM antibody glomerulonephritis [20]. It is presently unclear whether crescentic transformation of underlying membranous nephropathy implies an increase in the severity of the disease or development of a second process. Optimal treatment regimens need to be clarified and further research in this area is necessary.

## Consent

Written informed consent was obtained from the patient for publication of this case report and any accompanying images. A copy of the written consent is available for review by the Editor-in-Chief of this journal.

## Competing interests

The authors declare that they have no competing interests.

## Authors' contributions

DP made substantial contributions to conception and design, acquisition, analysis and interpretation of data. NN and AT were involved in drafting the manuscript or revising it critically for important intellectual content. All authors read and approved the final manuscript.
